# Triboelectric Energy Harvester Based on Stainless Steel/MoS_2_ and PET/ITO/PDMS for Potential Smart Healthcare Devices

**DOI:** 10.3390/nano11061533

**Published:** 2021-06-10

**Authors:** Carlos Gallardo-Vega, Octavio López-Lagunes, Omar I. Nava-Galindo, Arxel De León, Jorge Romero-García, Luz Antonio Aguilera-Cortés, Jaime Martínez-Castillo, Agustín L. Herrera-May

**Affiliations:** 1 Centro de Investigación en Química Aplicada, Saltillo 25294, Coahuila, Mexico; carlos.gallardo@ciqa.edu.mx (C.G.-V.); jorge.romero@ciqa.edu.mx (J.R.-G.); 2 Maestría en Ingeniería Aplicada, Facultad de Ingeniería de la Construcción y el Hábitat, Universidad Veracruzana, Boca del Río 94294, Veracruz, Mexico; olopzl@gmail.com; 3 Departamento de Ingeniería Mecánica, DICIS, Universidad de Guanajuato, Salamanca 36885, Guanajuato, Mexico; oi.navagalindo@ugto.mx (O.I.N.-G.); aguilera@ugto.mx (L.A.A.-C.); 4 CONACYT-Centro de Investigación en Química Aplicada, Saltillo 25294, Coahuila, Mexico; arxel.deleon@ciqa.edu.mx; 5 Micro and Nanotechnology Research Center, Universidad Veracruzana, Boca del Río 94294, Veracruz, Mexico; jaimartinez@uv.mx

**Keywords:** triboelectric energy harvesting, internet of things, molybdenum disulfide (MoS_2_), polydimethylsiloxane (PDMS), PET/ITO, smart healthcare device

## Abstract

The smart healthcare devices connected with the internet of things (IoT) for medical services can obtain physiological data of risk patients and communicate these data in real-time to doctors and hospitals. These devices require power sources with a sufficient lifetime to supply them energy, limiting the conventional electrochemical batteries. Additionally, these batteries may contain toxic materials that damage the health of patients and environment. An alternative solution to gradually substitute these electrochemical batteries is the development of triboelectric energy harvesters (TEHs), which can convert the kinetic energy of ambient into electrical energy. Here, we present the fabrication of a TEH formed by a stainless steel substrate (25 mm × 15 mm) coated with a molybdenum disulfide (MoS_2_) film as top element and a polydimethylsiloxane (PDMS) film deposited on indium tin oxide coated polyethylene terephthalate substrate (PET/ITO). This TEH has a generated maximum voltage of 2.3 V and maximum output power of 112.55 μW using a load resistance of 47 kΩ and a mechanical vibration to 59.7 Hz. The proposed TEH could be used to power potential smart healthcare devices.

## 1. Introduction

The smart healthcare devices connected to internet of things (IoT) can be used for continuous monitoring of the health of patients [1,2,3,4]. These devices with IoT allow the communication of several physiological data between patients and doctors [5,6,7,8,9]. Thus, these devices can be employed for monitoring the health of risk patients and taking immediately appropriate actions. The smart healthcare devices integrated with IoT could enhance the quality of the medical services to patients. In addition, these devices may be useful for healthcare in pandemic like COVID-19 [10,11,12,13]. Several smart healthcare devices are applied in clothes, wrist watches, bands and electronic-skin [14,15,16,17,18]. Most of these devices must contain materials with stretchable and wearable characteristics to be adjusted to different parts of the human body. In addition, novel wearable devices are demanding power between 1 and 100 μW in function of their size and electronic components [19]. Generally, these devices are powered using conventional electrochemical batteries that have a limited operation time and may contain some toxic materials [20]. These toxic components can generate environmental contamination and health damage. Thus, renewable power sources are necessary to power smart healthcare devices. For this, energy harvesters are green energy sources that can convert the ambient energy into electrical energy using transduction mechanisms such as thermoelectric, electromagnetic, piezoelectric and triboelectric [21,22,23,24]. The energy harvesters can use diode rectifiers to convert the alternating current (AC) in direct current (DC). In addition, these energy harvesters may use capacitors to store the electrical energy.

The triboelectric energy harvesters (TEHs) have important advantages to be used in smart healthcare devices, including flexible and wearable materials, simple design, low cost and easy fabrication process. Commonly, TEHs can scavenge energy from low frequency sources [25,26]. For instance, Zhang et al. [27] reported a porous micro-nickel foam (PMNF)-based TEH to scavenge natural vibration energy. This TEH (50 mm × 50 mm size) provides an open-circuit voltage and a short-circuit current of 187.8 V and 71.9 μA, respectively. Furthermore, the maximum output power of this TEH is 3.7 W/m^2^ at a frequency of 13.9 Hz. On the other hand, Ahmed et al. [28] developed a flexible and stretchable self-powered keyboard TEH based on urethane, silicone rubbers and carbon nanotubes (CNTs). This keyboard TEH can generate up to 2.5 V and a maximum output power of 0.3 μW. Zhang et al. [29] fabricated an expanded polytetrafluoroethylene (ePTFE)/Nylon-based TEH, which has a working area of 1 cm^2^. This ePTFE/Nylon TEH exhibits a maximum output power of 1.01 mW/cm^2^ at 10 Hz with a load resistance of 1 MΩ. Although of the great features of these TEHs, some of them have not simple designs and fabrication processes that can reduce their costs. Additionally, more investigations on TEHs using novel materials are required to improve their performance and decrease the fabrication costs [30,31]. Thus, future TEHs could be used in smart healthcare devices and wearable sensors. In this research topic, we propose a TEH composed by a top film of stainless steel/molybdenum disulfide (MoS_2_) and bottom film of polydimethylsiloxane (PDMS) with substrate of tin-doped indium oxide (ITO) coated polyethylene terephthalate (PET).

## 2. Materials and Methods

The materials used in the fabrication process of a TEH were as follows: ethanol (solvent 99.9%) and isopropyl alcohol (solvent 99.9%) were purchased by J.T. Baker (Houston, Texas, USA); and ITO/PET substrates, ammonium molybdate tetrahydrate (83%) and sodium sulfide hydrate (60%) were acquired from Sigma-Aldrich Chemical Company (St. Louis, MO, USA); PDMS (Sylgard 184) with a volume ratio of 10:1 was obtained from Dow Corning (Campbell, CA, USA).

The synthesis of MoS_2_ was implemented using a modified method of Li et al. [32] and posterior exfoliation with ultrasound. The first step for molybdenum disulfide synthesis was adding ammonium molybdate (250 mg) and sodium sulfide (47.35 mg) in 25 mL of water with a few drops to 0.1 M NaOH to pH 7. Then, the solution was mixed by the magnetic stirring machine (30 min). After the mixed solution was put into a teflon-lined stainless steel autoclave and left to react for 24 h at 180 °C, the product was then washed three times with ethanol by centrifugation (25 min at 13,000 rpm) and dried at RT. Subsequently, the molybdenum disulfide (2 gm) was added in isopropyl alcohol (100 mL) and then placed for a sonication process for 3 h at 30 amplitude. In order to scatter the MoS_2_ from the isopropyl alcohol, the sample was left to rest for a day. Finally, the precipitate (MoS_2_) was separated from the supernatant and then left to dry (RT). Finally, MoS_2_ (500 mg) and isopropyl alcohol (5 mL) were mixed to obtain a MoS_2_ dispersion by ultrasound for 30 min. Subsequently, the solution was spin coated (1 min at 100 rpm) on the stainless steel substrate and then dried at RT.

**Figure 1:**
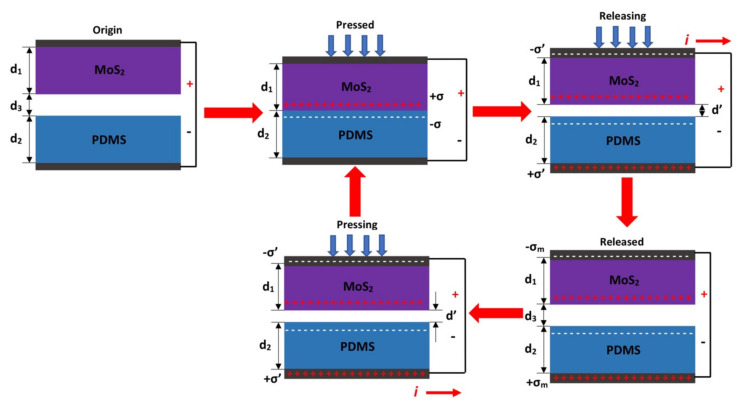
Schematic view of the fundamental operation in short-circuit condition of the TEH based on stainless steel/MoS_2_ and PET/ITO/PDMS.

The fundamental operation of TEH consists of the vertical contact separation of their layers, which is established by contact electrification and electrostatic induction. Figure 1 shows a schematic of the TEH, wherein two active materials and two electrodes are placed as a sandwich structure. The two active materials, MoS_2_ and PDMS are assigned as the top and bottom active materials, respectively. Each active material has a thickness, defined as d_1_ for the MoS_2_ and d_2_ for the PDMS, and a separation distance (d_3_) between them. In the origin position, the active materials has no generation nor induction of electrical charge. When the MoS_2_ and PDMS films are in contact due to an external force, electrons are transferred from the MoS_2_ surface to the PDMS surface. At the pressed state, these electrons generate a positive charge in the surface of MoS_2_ and a negative charge on the surface of PMDS. These charges are better known as triboelectric charges (σ). In the short-circuit condition, an induced charge (σ’) is generated once the top and bottom electrodes are connected, flowing a current among them. The induced positive and negative charges are accumulated on the top and bottom electrodes, respectively. Thus, a positive current is generated, and the original separation (d_3_) is changed to a small separation (d’) during the releasing state. At the released state, the maximum value of the induced charge (σ_m_) is obtained once the original separation distance (d_3_) is reached. Pressing the TEH again results in the reduction of d_3_, leaving the top electrode with a higher electrical potential than that of the lower electrode. Thus, electrons start to move from the bottom electrode to the top electrode, decreasing the number of induced charges and releasing a negative current. When the MoS_2_ and PDMS come back in contact, all the induced charges counteract each other.

**Figure 2:**
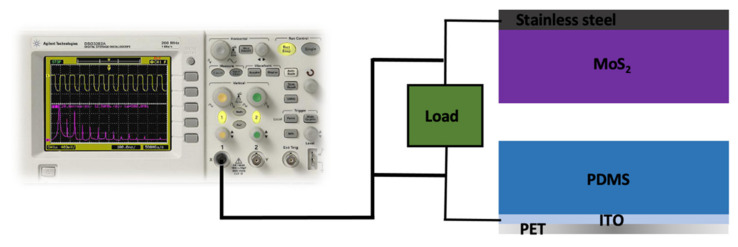
Schematic view of the experimental setup to measure the electrical response of the triboelectric energy harvester.

In the open circuit condition, the MoS_2_ surface is positively charged, and the PDMS surface is negatively charged. Once the relative positions of the MoS_2_ and PDMS films are altered, the electric potential difference is changed. Therefore, electrons move through the electrodes while the maximum electrical output is obtained. Figure 2 depicts a schematic view of the setup to measure the electrical response of the triboelectric energy harvester.

A large TEH area can increase the charges accumulated of its triboelectric layers, improving the output power [33]. However, the design of our TEH area was limited due to the working size of the spin coating machine. Thus, we used PET/ITO/PDMS and stainless steel/MoS_2_ films with a rectangular area of 25 mm × 15 mm. This area size is similar to that reported in other TEHs to harvest energy from human body motions [34,35,36,37,38,39].

The PET/ITO substrate was washed with ethanol three times using an ultrasonic bath (5 min). The stainless steel substrate was cleaned with chloroform into the ultrasonic washer for 5 min and 10 min. Later, two more times (5 min and 10 min) using ethanol. Finally, the substrates were dried using compressed air and carried out cleaning and drying at 25 °C.

**Figure 3:**
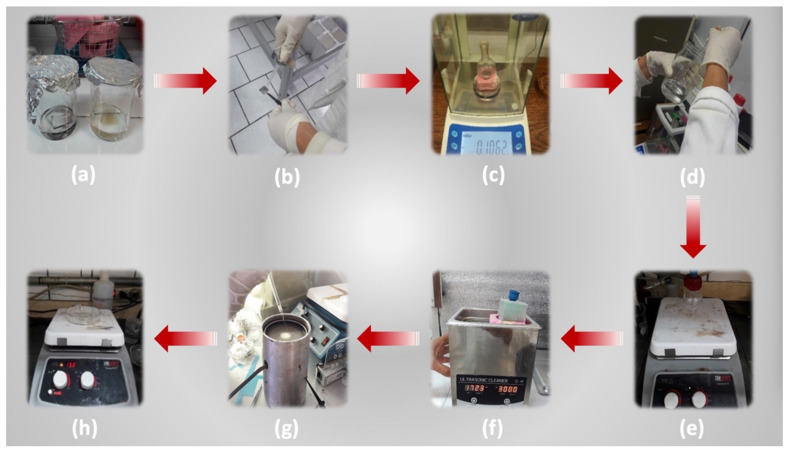
Images of the preparation procedure steps for the top and bottom films of the TEH: (**a**) substrates cleaning; (**b**) substrates drying; (**c**) samples weighing; (**d**) solution preparation; (**e**) magnetic stirring; (**f**) ultrasonic bathing; (**g**) spin coating; (**h**) drying.

On the other hand, the PDMS film was obtained by mixing the PDMS (100 mg) and curing agent (10 mg) in chloroform (3 mL). Later, PDMS (100 mg/mL) solution was mixed using the ultrasonic machine (30 min). Then, the PDMS film was coated on the PET/ITO substrate employing spin coating (1 min at 100 rpm) and heated in an oven for 1 h at 150 °C to PDMS crosslinking. Figure 3 shows the preparation procedure steps of the bottom and top films of the TEH.

**Figure 4:**
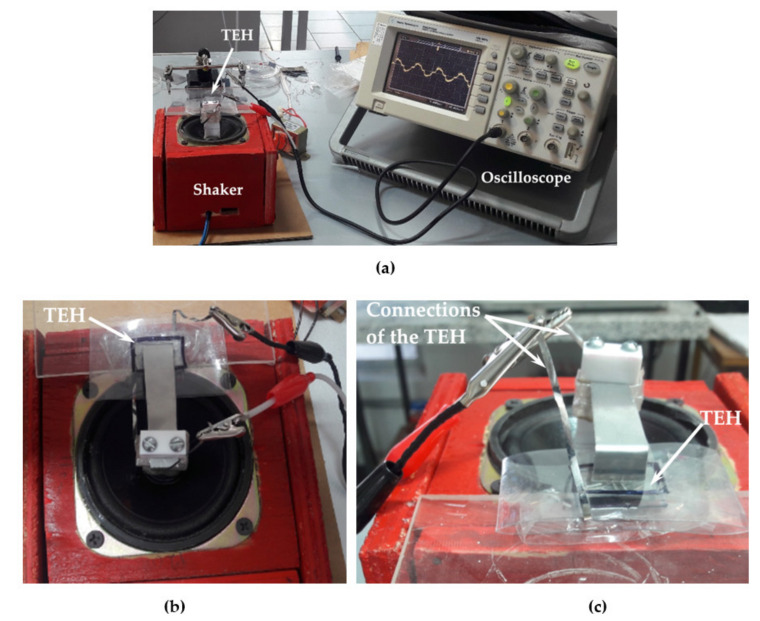
Experimental setup to measure the output voltage of the triboelectric energy harvester: (**a**) shaker, TEH and oscilloscope, (**b**) upper view of the TEH mounted on the shaker and (**c**) electrical connections of the TEH.

An electromagnetic shaker was constructed using a woofer coupled (CIQA, Saltillo, Coahuila, Mexico) to a signal amplifier TPA3118 (Texas Instruments, Dallas, TX, USA). A function generator (METEX model MXG-9810A) (METEX, Seoul, Korea) is employed to obtain the vibration amplitude and frequency of the shaker. The two layers of proposed TEH were mechanically excited using the shaker and its output voltage was measured using an oscilloscope (KEYSIGHT technologies model DSO3102A) (KEYSIGHT Technologies, Santa Rosa, CA, USA). Figure 4 shows the electrodes of the bottom and upper layers of the TEH connected to the oscilloscope.

The X-ray diffraction (XRD) study of MoS_2_ material was performed on an Eco D8 Advance of Bruker. The data were recorded in the range of 3–90° 2θ, at a rate 0.01°/min, 40 KV voltage and 25 mA of emission current. The MoS_2_ morphology was determined by transmission electron microscopy (TEM) with the FEI Titan microscope at 300 KV, by casting few drops of a dispersion of materials in isopropyl alcohol (0.1 mg/mL) on a Lacey carbon grid. AFM morphological study was performed on a Dimension™3100 from Digital Instruments with a Pt-coated Si tip with a 15 nm nominal radius model (OSCM-PT Bruker). The images were obtained at a scanning rate (256 lines) of 1 Hz.

## 3. Results and Discussion

**Figure 5:**
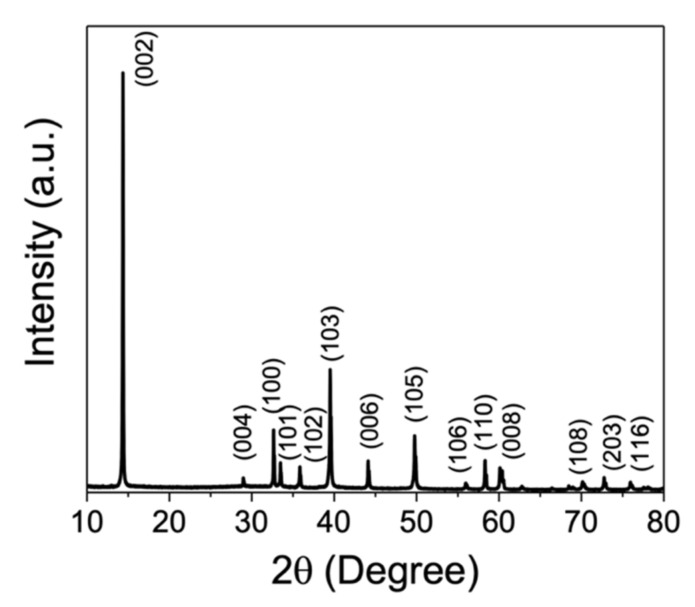
XRD result of MoS_2_ film of the triboelectric energy harvester.

The XRD analysis of MoS_2_ shows the reflection at 2θ = 14.3, 29.03, 32.6, 33.51, 35.87, 39.54, 44.15, 49.79, 55.98, 58,34, 60.15, 70.14 and 72.79 corresponded to MoS_2_ crystalline planes respectively: (002), (004), (100), (101), (102), (103), (006), (105), (106), (110), (008), (108) and (203) (JCDPS 37-1492). This result demonstrated the total conversion of the precursors (ammonium molybdate and sodium sulfide) into MoS_2_, without signals or other associated reflections for impurities (Figure 5).

**Figure 6:**
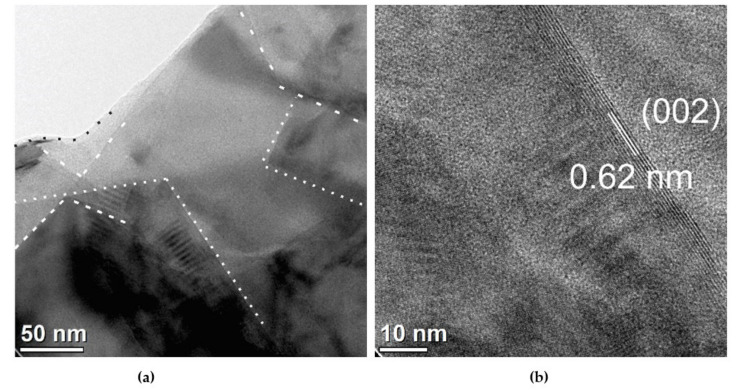
Morphological analysis of molybdenum disulfide film: (**a**) TEM and (**b**) HRTEM of the MoS_2_ crystals.

**Figure 7:**
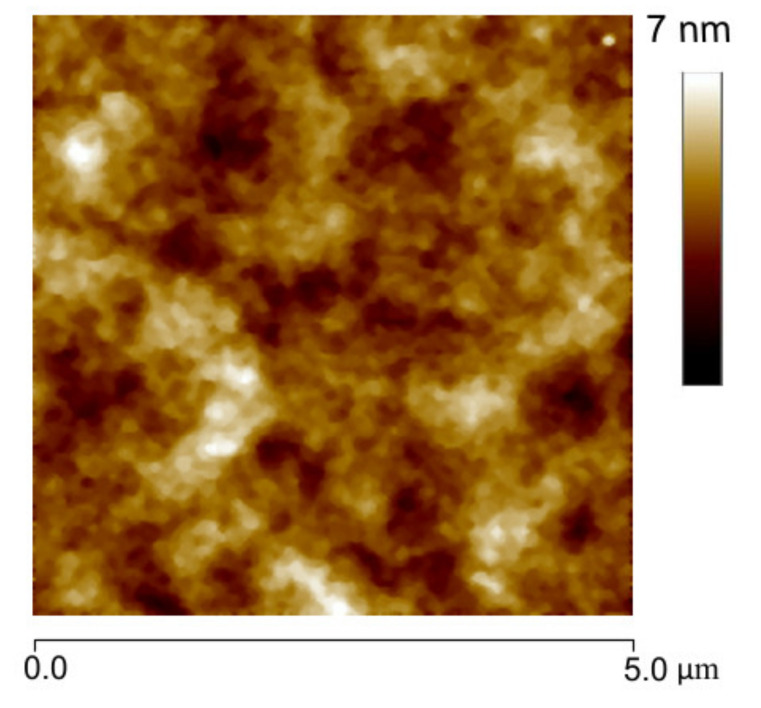
Morphological characteristics tapping image of PDMS film.

The morphological analysis was obtained by TEM (Figure 6), where we observed a few transparent and exfoliated layers of MoS_2_ (indicated as dashed regions), coexisting with stacked sheets (darkest regions). This indicate partial exfoliation for MoS_2_. The HRTEM shows the lattice spacing of 0.62 ± 0.002 that corresponds to (002) facet of MoS_2_ (JCDPS 37-1492). Figure 7 shows the morphological analysis of PDMS film by tapping mode. The PDMS presents uniform morphology with root mean square roughness Rq = 0.775 nm and roughness average Ra = 0.611 nm.

**Figure 8:**
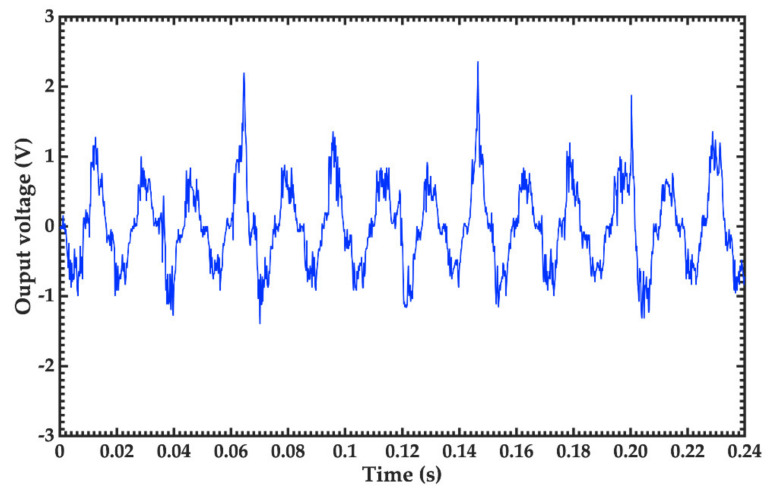
Measurements of the output voltage of the triboelectric energy harvester using a load resistance of 47 kΩ and a mechanical excitation at 59.7 Hz.

Next, we connected to the TEH a load resistance of 47 kΩ to measure its output voltage. For this, a mechanical vibration to 59.7 Hz was applied. Figure 8 depicts the peak-peak output voltage of the TEH during 0.24 s. Based on this response, the maximum output voltage of the TEH is close to 2.3 V. Thus, the maximum output power and power density are 112.55 μW and 30 μW/cm^2^, respectively. This power overcomes the range from 1 to 100 μW that demands several smart healthcare devices reported in the literature [19]. However, future works must include more investigations about performance and reliability of TEHs based on stainless steel/MoS_2_ and PET/ITO/PDMS.

Table 1 depicts the main parameters of the performance of our TEH device in comparison with other devices that include MoS_2_ films. These parameters consider the materials films, contact area, test frequency, resistance, voltage and power density. Our TEH shows good performance based on a simple and low-cost fabrication process.

## 4. Conclusions

A TEH integrated by stainless steel/MoS_2_ and PET/ITO/PDMS films was presented. This TEH was developed using a simple and low-cost fabrication process. XRD and morphological analysis of the MoS_2_ film were implemented. In addition, morphological characteristics of PDMS film was investigated using AFM. The electrical response of the proposed energy harvester was measured applying a mechanical excitation to 59.7 Hz and a load resistance of 47 kΩ. The maximum voltage and power density of the TEH were 2.3 V and 30 μW/cm^2^, respectively. This electrical performance of the energy harvester could allow its potential application into smart healthcare device.

## Figures and Tables

**Table 1:** Comparison of the main parameters of different TEH devices that contain MoS_2_ film.

TEH Structure Materials	Contact Area (cm^2^)	Frequency (Hz)	Resistance (Ω)	Voltage (V)	Power Density (μW/cm^2^)	Ref.
PET/Al/PI Glass/Al/PI/MoS_2_:PI/PI	3.75	5	5 × 10^6^	120	2570	[40]
Cu/PVDF/MoS_2_-cellulose paper/PVDF/Cu Cu/Polyimide	9	—	1 × 10^6^	50	180	[41]
PET/PPy PET/ITO/PDMS/PVDF-ferroelectric/MoS_2_	1	10	10 × 10^6^	-80	455	[42]
Al-MoS_2_-Glue/Paper Al/Graphite-Glue/Paper	9	3	3.2 × 10^6^	3.82	7.44 × 10^−2^	[43]
ITO:PET/PVDF-TrFE/MoS_2_ ITO:PET/Nylon11/MoS_2_	1	6.5	10 × 10^6^	145	5 × 10^4^	[44]
Steel-MoS_2_ PET/ITO/PDMS	3.75	59.7	47 × 10^3^	2.3	30	Our work

Note: PI is polymide.

## Data Availability

Request the corresponding author of this article.
